# Anti-tumor activity of N-trimethyl chitosan-encapsulated camptothecin in a mouse melanoma model

**DOI:** 10.1186/1756-9966-29-76

**Published:** 2010-06-17

**Authors:** Xian-ping Liu, Sheng-tao Zhou, Xing-yi Li, Xian-cheng Chen, Xia Zhao, Zhi-yong Qian, Li-na Zhou, Zhi-yong Li, Yu-mei Wang, Qian Zhong, Tao Yi, Zheng-yu Li, Xiang He, Yu-quan Wei

**Affiliations:** 1 Department of Gynecology and Obstetrics, West China Second Hospital, Sichuan University, Chengdu 610041, China; 2 State Key Laboratory, Biotherapy and Cancer Center, West China Hospital, Sichuan University, Chengdu 610041, China

## Abstract

**Background:**

Camptothecin (CPT) has recently attracted increasing attention as a promising anticancer agent for a variety of tumors. But the clinical application is largely hampered by its extreme water insolubility and unpredictable side effect. It is essential to establish an efficient and safe protocol for the administration of CPT versus melanoma.

**Methods:**

Camptothecin was encapsulated with N-trimethyl chitosan (CPT-TMC) through microprecipitation and sonication. Its inhibition effect on B16-F10 cell proliferation and induction of apoptosis was evaluated by MTT assay and flow cytometric analysis in vitro. The anti-tumor activity of CPT-TMC was evaluated in C57BL/6 mice bearing B16-F10 melanoma. Tumor volume, tumor weight and survival time were recorded. Assessment of apoptotic cells within tumor tissue was performed by TUNEL assay. Antiangiogenesis and antiproliferation effects of CPT-TMC in vivo were conducted via CD31 and PCNA immunohistochemistry, respectively.

**Results:**

CPT-TMC efficiently inhibited B16-F10 cells proliferation and increased apoptosis in vitro. Experiment group showed significant inhibition compared with free CPT-treated group (81.3% vs. 56.9%) in the growth of B16-F10 melanoma xenografts and prolonged the survival time of the treated mice (P < 0.05). Decreased cell proliferation, increased tumor apoptosis as well as a reduction in angiogenesis were observed.

**Conclusions:**

Our data suggest that N-trimethyl chitosan-encapsulated camptothecin is superior to free CPT by overcoming its insolubility and finally raises the potential of its application in melanoma therapy.

## Background

Camptothecin (CPT) is an alkaloid isolated from the stem of the tree *Camptotheca acuminata *with its chemical structure identified by Wall et al. in 1966 [1] for the first time. It has a high anti-tumor activity in a wide range of cancers, such as colon, ovarian, breast, melanoma, lung and pancreatic cancers [2-6]. However, its poor water solubility, low stability in physiological medium and indefinite severe toxicity limite its further clinical application. Therefore, finding a novel drug delivery system is imperative to overcome these internal defects and to increase the anticancer efficacy of CPT currently [7].

In recent years, chitosan, a natural biomateria 1 obtained by hydrolyzing chitin has been exerted more and more emphasis in the fields of biomedical materials for delaying the drugs release and favorable biological properties including biocompatibility, biodegradability and nontoxicity [8,9]. However, the fact that chitosan is only soluble in an environment with pH values lower than 6.0 compromised its practical value in the pharmaceutical field. N-trimethyl chitosan (TMC), a derivate of chitosan, solves this problem. Compared with chitosan, TMC is soluble in the entire pH range. As a nonabsorbable and nontoxic polymers, TMC have also been confirmed to effectively ameliorate the permeation of hydrophilic macromolecules across mucosal epithelia by opening the intercellular tight junctions [10], thereby favoring the paracellular transport of drugs. In addition, this chitosan derivation possesses excellent drug loading capability and is a superior pharmaceutical excipients for drug delivery, which might serve as an available drug carrier to encapsulate camptothecin and facilitate the uptake and retention of camptothecin in cancers.

Melanoma mostly originates in epidermal melanocytes. It often occurs in the skin but could also be found in pigmented ocular structures, mainly in the uvea (choroid, iris and ciliary body), the gastrointestinal tract, soft brain (spinal) film, mouth and genital mucosa. The incidence of malignant melanoma accounts for only 5% of all skin cancer, but is increasing year after year worldwide and causes the largest number of skin cancer-related deaths worldwide, 3 times of all the other skin cancers, accounting for 75% [11]. It is characterized by strong invasiveness, high metastasis rate, rapid progression, and poor prognosis. Currently the treatments for melanoma include surgery resection, radiotherapy, chemotherapy, immunotherapy and biological therapy, usually with severe side effects [12]. Especially, some patients may develop relapse and metastasis or even die after treatment. Therefore, it is urgently needed to develop a more reliable and less toxic strategy to fight melanoma.

Previously, our laboratory encapsulated camptothecin with N-trimethyl chitosan and tested its anti-tumor efficacy in a mouse B16-F10 melanoma model. The present study was aimed to verify whether the new protocol could be more efficient and less toxic in melanoma treatment.

## Methods

### Cell culture and reagents

B16-F10 mouse melanoma cell lines were purchased from the American Type Culture Collection (ATCC, Rockville MD, USA) and preserved by the State Key Laboratory of Biotherapy of Human Diseases (West China Hospital of Sichuan University, Chengdu, People's Republic of China). Cells were cultured in RPMI1640 medium (Gibico BRL, Grand Island, NY, USA) supplemented with 10% fetal bovine serum(FBS) plus 100 μg/ml amikacin in a 37°C humidified chamber containing 5% CO2.

### Preparation of camptothecine nanoparticle (CPT-TMC)

CPT-TMC was prepared by combination of microprecipitation and sonication as follows: Firstly, 6 mg/ml of camptothecine was prepared by dissolving 30 mg camptothecine into 5 ml dimethyl sulfoxide (DMSO) solution. Then TMC was dissolved in water at the concentration of 5 mg/ml. Subsequently, 0.1 ml of camptothecine solution was added dropwisely into 2 ml of TMC solution at 4°C. The obtained colloid solution was ultrasonicated for 10 min also at 4°C. Finally, the colloid solution was dialyzed against water using a membrane with a molecular weight cutoff of 8,000-14,000 (Solarbio, China) for 3 days, then the solution was centrifuged at 10,000 × g for 10 min to remove insoluble CPT. The encapsulation rate of CPT to TMC was about 10% in this paper. The prepared CPT nanoparticles are well-dispersed and physical stable at 5 mg/ml TMC solution. The morphology of resulting CPT nanoparticles was investigated by transmission electron microscopy (TEM) observation. We could find that the needle-liked CPT nanoparticles were successfully prepared. The chiastic size of nanoparticles was only about 30-50 nm and vertical size of nanoparticles was about 500 nm. The zeta potential of resulting CPT nanoparticles was about +15 mv. CPT-TMC, CPT and TMC were dissolved in 0.9% NaCl solution (NS) for vitro and vivo studies.

### Inhibition of proliferation in vitro

MTT assay was applied to investigate the inhibition effect of CPT-TMC on B16-F10 cells proliferation. Medium with CPT-TMC, CPT and TMC were prepared respectively at same concentration. Each type of medium was further diluted into a series of 1/2 dilutions in six tubes (from 0.1 μg/ml to 3.2 μg/ml). Each dilution was added into triplicate wells of B16-F10 cells seeded on 96-well plates on the previous day (3 × 10^3 ^cells in complete medium per well). The cells were incubated at 37°C in 5% CO_2 _for 48 hours. Then, each well received 20 μl MTT solution (5 mg/ml). After a 3-hour incubation, the medium were removed and 150 μl DMSO were added. We put the plate in a shaker before reading absorbance at 490 nm using a microplate reader (3550-UV, BIO-RAD, USA) [13] after 20 min of incubation. The procedure was repeated three times with similar results. The following formula was used to calculate the inhibition rate of B16-F10 cells proliferation: (1- experimental group OD value/negative control OD value) ×100%. Media-only treated (untreated) cells were considered as the negative control group.

### Apoptosis assay in vitro

Quantitative evaluation of cellular apoptosis was performed by flow cytometric. Briefly, 2.5 × 10^5 ^B16-F10 cells were seeded in six-well plates and grew for 24 h to 70% confluence. Then cells were incubated with CPT-TMC, CPT, TMC at a concentration of 0.4 μg/ml, or media-only for another 48 h, respectively. After processed as described above, the floated cells were discarded while the attached cells were trypsinized and thereafter washed twice with cold PBS. Then cells were resuspended in prediluted binding buffer. Propidium iodide (PI, 1 μg/ml) was added, and the mixtures were immediately analyzed on an EPICS Elite ESP flow cytometer (Beckman Coulter, Hialeah, Fla., USA).

### Animal model and study design

The studies involving mice were approved by the Institutional Animal Care and Use Committee of Sichuan University (Chengdu, Sichuan, People's Republic of China). Female C57BL/6 mice, 6 to 8 weeks old, nonfertile, were purchased from the West China Experimental Animal Center of Sichuan University (Sichuan, China), and were maintained in pathogen-free conditions with sterile chow. 1 × 10^5 ^B16-F10 melanoma cells resuspended in 0.05 ml of PBS were injected subcutaneously into the right flank of each mouse. 9 days after injection when most of the tumors were palpable, the tumor-bearing mice were randomly divided into four groups (10/group): (a) mice treated with CPT-TMC (2.5 mg/kg), (b) mice treated with CPT (2.5 mg/kg), (c) mice treated with TMC (25 mg/kg), and (d) mice treated with 0.9% NaCl solution (NS,10 ml). Treatments were performed twice weekly for 2 weeks. Tumor sizes were measured every 3 days and were calculated using the formula A × B^2 ^× 0.52 (A, length; B, width; all measured in millimeters) [14]. When any mice began to moribund they were sacrificed. Subcutaneous tumors from sacrificed mice were removed and fixed in 4% paraformaldehyde solution for immunochemistry staining.

### Immunohistochemical assay

Tumors fixed in 4% paraformaldehyde solution were embedded in paraffin and sliced into 5 μm sections for tumor cell proliferation and microvessel density (MVD) quantification with proliferating cell nuclear antigen (PCNA) and CD31 immunohistochemistry respectively by the method reported by Weidner et al [15]. PCNA specifically expressed in the proliferating cell nucleus and the positive cells presented brown nuclei. PCNA immunostaining was used to assess tumor cell proliferation. CD31 had high affinity specific to vascular endothelial cell with brown-staining by biotinylation under microscopy. CD31 vessel immunostaining was performed to assess the angiogenesis in tumor tissues. Microvessel that presented brown-staining endothelial cell or endothelial cell cluster was considered as a countable microvessel.

The procedure was previously described in detail [16]. Sections were deparaffinized and rehydrated, followed by antigen retrieval with retrieval buffer (10 mmol/l pH 6.0 EDTA citrate buffer; Dako, Glostrup, Denmark). The peroxidase activity was inhibited by 3% H_2_O_2 _and the sections were incubated with 10% normal goat serum to blocking the non-specific binding of reagents. Rat anti-mouse CD31 antibody (1:100, Santa Cruz Biotechnology) and mouse anti-human PCNA antibody (1: 100, Santa Cruz Biotechnology) were applied as primary antibody overnight in a moist chamber at 4°C. Goat anti-rat immunoglobulin (1:100, Santa Cruz Biotechnology) and goat anti-mouse immunoglobulin (1:100, Santa Cruz Biotechnology)were applied as secondary antibody for 40 min at 37°C, followed by the streptavidin-biotin complex method. Immunostaining was developed using DAKO Liquid DAB+ Substrate-Chromogen System (ZSJQ Biotechnology, Beijing, China), followed by counterstaining with hematoxylin.

Image of tumor tissue was taken by using OLYMPUS BX600 microscope and SPOT FIEX camera.

### TUNEL detection

Analysis of apoptotic cells in tumor tissue was performed by Terminal deoxynucleotidyl transferase-mediated dUTP nick-end labeling (TUNEL) staining using an apoptotic cell detection kit following the manufacturer's directions (Promega, Madison, Wisc., USA). TUNEL-positive cells had pyknotic nucleus with dark green fluorescent staining, pointed apoptosis. Images of the sections were taken by a fluorescence microscope (Olympus, Tokyo, Japan). Apoptosis index was calculated by dividing the number of TUNEL-positive cells by the total number of cells in the field.

### Evaluation of possible side effects

Mice, especially those treated with CPT-TMC, had been observed for potential side effects through weight, appetite, diarrhea, life span, and behavior until they were sacrificed. Organs such as heart, liver, spleen, lung, and kidney were collected and made into 5 μm sections which were stained with hematoxylin and eosin (H&E) and observed under a microscope.

### Statistical analysis

One-way analysis of variance (ANOVA) was used to determine statistical significances in comparisons of MTT assay, tumor volume, animal weight, tumor weight, microvessel density (MVD), PCNA immunostaining and TUNEL assay among different groups. Comparisons of survival curves were based on the Kaplan-Meier method and Log-rank test was used to compare survival rate. P < 0.05 was considered statistically significant.

## Results

### CPT-TMC inhibited cell proliferation and promoted apoptosis in vitro

**Figure 1:**
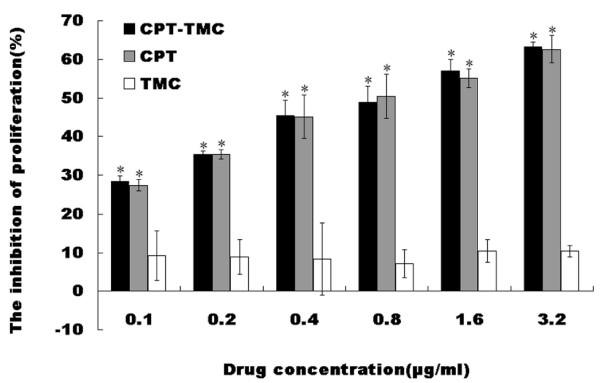
**Inhibitory effect of CPT-TMC on B16-F10 cells proliferation in vitro**. The proliferation of B16-F10 cells was assessed by the MTT assay. Data were assessed as percent cell viability in terms of media-only treated (non-treated) control cells at each drug concentration. It is clear that CPT-TMC caused a dose-dependent inhibition of proliferation in vitro. Means ± SD (n = 3). *P < 0.05

B16-F10 cell proliferation was examined using the MTT assay. As shown in Fig. 1, CPT-TMC and CPT significantly reduced the proliferation of B16-F10 cells compared with TMC and media-only (*P < 0.05). Their inhibitory rate increased in a concentration-dependent manner. However, no significant difference was observed between CPT-TMC and CPT group, as well as TMC and media-only group (P > 0.05).

**Figure 2:**
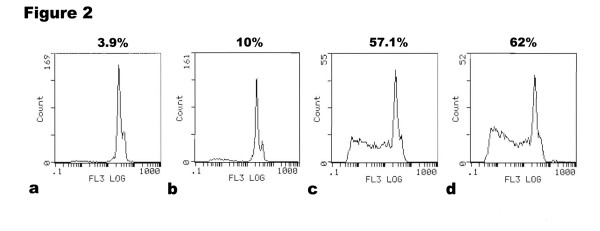
**Induction of apoptosis on B16-F10 cells by CPT-TMC in vitro**. Cellular apoptosis was verified by flow cytometric analysis. B16-F10 Cells were treated with (a) media-only, (b) TMC, (c) CPT, or (d) CPT-TMC, respectively. It is clear that the number of apoptotic cells in CPT-TMC and CPT treated group was significantly higher compared with other two groups. The apoptotic rate showed 62% in CPT-TMC-treated group versus 57.1% in CPT-treated group, 10% in TMC-treated group and 3.9% in media-only-treated group.

Furthermore, it was evaluated by flow cytometry whether the inhibition in cell proliferation resulted from apoptosis induction. The numbers of apoptotic cells in CPT-TMC and CPT treated group were significantly higher compared with other two groups. The apoptotic rate showed 62% in CPT-TMC-treated group versus 57.1% in CPT-treated group, 10% in TMC-treated group and 3.9% in media-only-treated group (Fig. 2). Results obtained from flow cytometry strongly correlated with the MTT assay data.

### CPT-TMC inhibited tumor growth in vivo

**Figure 3:**
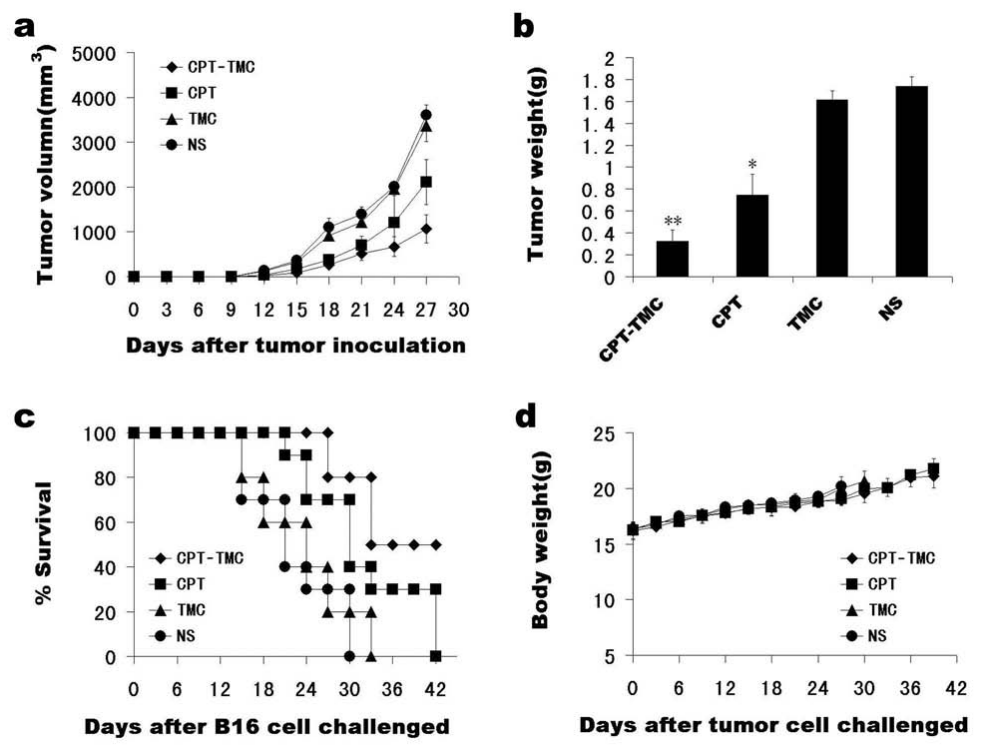
**Anti-tumor efficacy of CPT-TMC in vivo**. The tumor models were established in C57/BL6 mice (10/group) and then were treated with i.v. administration of 2.5 mg/kg CPT-TMC, 2.5 mg/kg free CPT, 25 mg/kg TMC, or NS twice per week, when tumors were palpable. (a) Tumor volume growth curve. Tumor sizes were measured every 3 days. CPT-TMC significantly inhibited tumor growth. There was a significant difference in tumor volume between CPT-TMC and control groups (P < 0.05). (b) Comparison of the tumor weight. At the third day after the last treatment, mice were sacrificed, and tumors were removed and weighed. Significant differences between CPT-TMC group and control groups are represented (*P < 0.05, **P < 0.01). Values are means ± SD. (c) Survival curve for tumor-bearing mice. A significant increase in survival in CPT-TMC-treated mice was also found when compared with the control groups (P < 0.05, by Log-rank test). And there was no statistical difference between TMC-treated mice and NS-treated mice (P > 0.05). (d) Lack of toxicity-dependent weight loss in tumor-bearing mice treated with CPT-TMC. There are no significant differences in weight among the four groups (P > 0.05). Values are means ± SD.

Tumor volume in CPT-TMC-treated group was significant smaller than control groups (P < 0.05). Mean tumor volume (± SD) in CPT-TMC-treated mice was 1067 ± 311 mm^3 ^versus 2108 ± 502 mm^3 ^in CPT-treated mice, 3367 ± 353 mm^3 ^in TMC-treated mice and 3607 ± 220 mm^3 ^in NS-treated mice (Fig. 3a). Although tumor volume in TMC-treated group is smaller than NS-treated group, there was no significant difference between them, P > 0.05. Tumor weight was measured on the third day after the last treatment. Mean tumor weight was 0.324 ± 0.101 g, 0.748 ± 0.186 g, 1.616 ± 0.079 g and 1.736 ± 0.087 g in CPT-TMC, CPT, TMC and NS treated group, respectively (Fig. 3b).

### CPT-TMC prolonged survival of tumor-bearing mice

Survival of CPT-TMC group was significantly prolonged compared with controls, P < 0.05. As shown in Fig. 3c, NS-treated group showed 0% survival on day 30, TMC-treated group showed 0% survival on day 33, and CPT-treated group showed 0% survival on day 42. In contrast, CPT-TMC-treated group had a 50% survival rate persisting up to day 42. The 0% survival of the CPT-TMC-treated group happened on the day 51.

### Toxicity observation

We measured the animal weight every 3 days and found no significant difference among the four groups (Fig. 3d). We also considered appetite, fur, behavior etc. for evaluation of physical status and there were no changes in gross measures. In addition, H&E histological staining of the heart, liver, spleen, lung, and kidney indicated no significant differences between CPT-TMC-treated and the control mice.

### CPT-TMC inhibited cell proliferation in vivo

**Figure 4:**
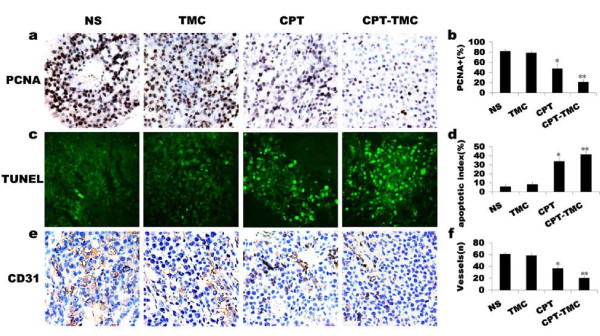
**CD31, PCNA and TUNEL analyses for tumor tissue**. (a) Tumor sections immunostained with an antibody against PCNA revealed that there were many strongly positive nuclei in control tumor tissues, whereas such nuclei were rare in tumor tissues of CPT-TMC-treated group. (b) Quantification of PCNA staining showed percentage of PCNA-positive nuclei in CPT-TMC-treated group was the lowest among the four groups (*P < 0.05, **P < 0.01). (c) Apoptosis of tumor tissues in different groups were calculated by TUNEL assays, which showed that CPT-TMC induced a significant enhancement of apoptotic cells in contrast to control therapies. (d) Quantification of TUNEL assay shows that apoptosis index of CPT-TMC-treated tumor was much higher than that of control groups (*P < 0.05, **P < 0.01). (e) Tumor sections immunostained with anti-CD31 antibody (brown) for angiogenesis assay. Representative sections were taken from tumor tissue of NS-treated, TMC-treated, CPT-treated and CPT-TMC-treated groups. (f) Histomorphometric assay for tumor microvessels revealed that MVD was significantly lower in CPT-TMC-treated group compared with the controls (*P < 0.05, **P < 0.01).

Because CPT-TMC inhibited cell proliferation obviously in vitro, we first examined its effects on tumor cell proliferation by PCNA staining to explore the potential mechanisms of CPT-TMC therapy in vivo. PCNA expression was apparently reduced in CPT-TMC-treated group compared with other groups (Fig. 4a). Our data showed the percentage of PCNA-positive cells was 21.4 ± 4.3% in CPT-TMC-treated tumors versus 47.4 ± 9.4% in CPT-treated tumors, 78.8 ± 3.4% in TMC-treated tumors and 81.8 ± 3.1% in NS-treated tumors, respectively (Fig. 4b).

### CPT-TMC increased intratumoral apoptosis

TUNEL assay was performed to detect tumor cell apoptosis to further investigate the role of CPT-TMC treatment in tumor in vivo. As shown in Fig. 4c, CPT-TMC-treated tumors showed significantly more apoptotic cells (with green nuclei) than tumors from CPT, TMC or NS treated groups. The apoptosis index was significantly higher in CPT-TMC-treated group compared with the controls (**P < 0.01): Mean apoptotic index ± SD of tumor cells treated with CPT-TMC was 41.4 ± 2.8% when it was 34 ± 3.9%, 8.2 ± 2.2%, or 5.8 ± 1.6% in CPT, TMC, or NS treated group, respectively (Fig. 4d). These results suggested that the increased tumor cell apoptosis by CPT-TMC treatment in vivo may explain why tumor volumes shrinked.

### CPT-TMC inhibited intratumoral angiogenesis

Anti-angiogenesis is a major anticancer mechanism. Therefore, MVD was evaluated in the tumors by counting the number of microvessels in sections stained with CD31 to further investigate the anti-angiogenic effect of CPT-TMC. CD31-positive single or a cluster of cells were counted as the microvessels (Fig. 4e). As shown in Fig. 4f, MVD reduced the most significantly in CPT-TMC-treated group (20.4 ± 2.9) compared with CPT (36.8 ± 2.5), TMC (58.8 ± 2.9) and NS treatments (61 ± 2; **P < 0.01). No significant difference was found between TMC group and NS group (P > 0.05). The inhibition of tumor neovascularization after CPT-TMC treatment may partially explain the apoptosis induction which subsequently reduce tumor progression and finally prolong survival time.

## Discussion

Nanoparticles may be defined as submicronic colloidal systems that are generally composed of polymers. In recent years, nanoparticles have been explored with some success in maintaining or improving the anti-tumor activity of the anticancer agents. Nanoparticles can penetrate into the membrane cells and spread along the nerve synapses, blood vessels and lymphatic vessels, with the capacity of selectively accumulating in different cells and certain cell structures at the same time. The formulation of nanoparticles and physicochemical parameters such as pH, surface charge are critical for drug delivery. The interaction of drug carrier systems with the biological environment is important for designing strategies: these systems should be independent in the environment and selective at the pharmacological site. If designed appropriately, nanoparticles may act as a powerful drug vehicle able to target tumor tissues or cells and prevent the drug from inactivation during its transportation.

The selection of agents as drug delivery system is essential in the process of nanoparticle preparation for drug delivery system. Chitosan is renowned for its function of drug and gene delivery to cells and tissues [17,18]. The medical materials made of chitosan, not only possess the characteristics of the general physicochemical polymer materials, such as mechanical stability and acceptability to sterilization, but also can be transformed into small molecular substances. Furthermore, they can also be easily absorbed by enzymatic hydrolysis, thereby with no toxic effects in vivo. However, chitosan could only dissolve in acidic environments, compromising its application prospect. N-trimethyl chitosan (TMC), a derivative of chitosan with cation, is soluble within a wide pH range. It can interact with the negative charge and tight junctions on the cell surface, and afterwards open the tight junctions between cells [19]. Due to its good biocompatibility, biodegradability, hydrophilicity and bio-adhesion, TMC as a vascular targeting vector for anti-tumor chemotherapy drugs, has superior to other synthetic vectors, such as the toxic cationic lipid materials. Therefore, in recent years, TMC has been widely used in drug targeting delivery systems [20-22].

Camptothecin, a component of the stem of the tree *Camptotheca acuminata *extracts, is known for its efficient anti-tumor activity. It has multiple pharmacologic actions including anti-angiogenesis, anti-tumor, immunosuppression, anti-virus, and anti-early pregnancy. A large number of studies have revealed that camptothecin can induce apoptosis in leukemia, colon cancer, prostate cancer and other tumor cells. Despite the common clinical use of camptothecin or its derivatives for the treatment of cancers, its poor solubility still remains to be resolved. In addition, because the lactone ring of camptothecin and its derivatives is unstable in the presence of human serum albumin, the active drug often easily changes into inactive carboxylate form bound to albumin [23]. The low stability of camptothecin hampers its delivery capability to the tumor to reach an effective concentration. The selective increase in tumor tissue uptake of anticancer agents would be of great interest.

Cengelli F, et al [24] covalently linked camptothecin to biocompatible ultrasmall superparamagnetic iron oxide nanoparticles (USPIOs) coated with polyvinylalcohol/polyvinylamine (PVA/aminoPVA). These CPT-USPIO conjugates exhibited antiproliferative activity in vitro against human melanoma cells. Huang ZR, et al [25] prepared lipid nanoparticles made of Precirol (solid lipid nanoparticles; SLN-P), Compritol (SLN-C), Precirol+squalene (nanostructured lipid carriers; NLC), and squalene (a lipid emulsion; LE). No superiority for camptothecin in cytotoxic activities in vitro was found except for camptothecin loaded in the SLN-P. However, both of the two researchers didn't use their camptothecin nanoparticles in vivo study. Loch-Neckel G, et al[26] evaluated the effect of intraperitoneally administered methoxy polyethylene glycol-(D,L-lactide) (PLA-PEG) (49 and 66.6 kDa) and Poly (D,L-lactide) PLA nanocapsules containing CPT on lung metastatic spread in mice inoculated with B16-F10 melanoma cells, and on the cytotoxic activity against B16-F10 melanoma cells in vitro. In vitro study, both PLA and 49 kDa PLA-PEG nanocapsules containing CPT were more cytotoxic than the free CPT against B16-F10 melanoma cells. Only CPT-loaded PLA-PEG 49 kD nanocapsules significantly decreased the number of lung metastases when compared with free drug.

Although there were some reports about encapsulating camptothecin in nanoparticles as a potential antiproliferative treatment for cancer before, this study is the first research that encapsulated camptothecin with N-trimethyl chitosan by combination of microprecipitation and sonication, and examined it in a mouse melanoma model. Using this feasible model, we can investigate the local tumor growth inhibition by CPT-TMC.

Tumor blood vessels apt to expand compared with physiological vessels. The rapidly expanding tumor vasculature often has a discontinuous endothelium, with gaps between the cells that may be several hundred nanometers large [27,28]. We encapsulated camptothecin with N-trimethyl chitosan, and the nanoparticles may be targeted to the particulate region of capillary endothelium. Nanoparticles loaded with anticancer agents can successfully increase drug concentration in cancer tissues and decrease drug concentration in other normal tissues, and then enhance anti-tumor efficacy and improve the safety of CPT. N-trimethyl chitosan can provide controlled and targeted delivery of camptothecin with better efficacy.

The effect of CPT-TMC on B16-F10 cells was explored in vitro. Results showed that both CPT-TMC and CPT significantly inhibited B16-F10 cells proliferation and induced apoptosis while TMC showed no similar effect. No significant difference was found in the MTT assay between CPT and CPT-TMC. The possible reason for the lack of difference is that the pharmacologically important lactone ring of camptothecin is unstable in the presence of serum albumin which results in the conversion of the active drug to the inactive carboxylate form bound to albumin while there is no serum albumin in vitro to do so. In an attempt to overcome the disadvantage we encapsulated camptothecin with N-trimethyl chitosan and the results showed that camptothecin nanoparticle is superiority in vivo rather than in vitro.

We applied the CPT-TMC on a mouse melanoma model. As expected, CPT-TMC efficiently inhibited the growth of B16-F10 cancer xenografts, and significantly prolonged the survival time of the treated mice, while CPT only partially inhibited tumor growth. It may be explained that there was a temporary high serum but low intratumor levels of CPT because of nonselective expression and subsequent elimination. CPT-TMC showed significant suppression of tumor growth with the drug administered in the dose and schedule under the conditions of our study, causing no gross toxicity of the animals. In contrast, there was no significant difference in tumor volume and survival time between TMC-treated and NS-treated mice. Hence, CPT-TMC is a more tumor-specific approach, enhancing the therapeutic efficacy on tumor.

To elucidate the anti-tumor mechanism of CPT-TMC in vivo, proliferation, apoptosis and angiogenesis were systematically analyzed. Immunohistochemistry staining with PCNA and TUNEL assay of tumor tissue were performed. Our findings were consistent with the anti-proliferation and apoptosis-inducing ability of camptothecin mentioned above. The quantitative analysis showed that CPT-TMC-treated group had a significant reduction of PCNA-positive cells and increment of apoptotic index in contrast to other groups. Accumulated evidence indicates that a nascent tumor can stimulate angiogenesis. Angiogenesis plays a vital role in tumor growth. When a tumor grows to 1-2 mm, tumor cells have to depend on newborn vessels to provide oxygen and nutrients [29]. Hence, anti-angiogenic therapy has been considered to be a new direction to fight cancers [30-34]. When angiogenesis is inhibited, the supported tumor cells by those vessels subsequently suffer apoptosis [35]. Treatment with CPT-TMC resulted in apparent reduction in intratumoral MVD of melanoma compared with controls.

In summary, we demonstrated that CPT-TMC exerted anti-tumor activity through inhibiting cells proliferation, increasing apoptosis and reducing MVD. It may suggest that CPT-TMC was more effective than single CPT treatment. No significant difference in the percentage of PCNA- and TUNEL-positive cells, as well as MVD was found between the TMC and NS groups, suggesting that the control vector only posed minor impact on the anti-tumor effects and little toxicity to cells in vivo. These results strongly demonstrated that CPT-TMC may be an efficient and safe protocol for the administration of CPT versus melanoma.

## Conclusions

In conclusion, being encapsulated with N-trimethyl chitosan made camptothecin more efficacious against mouse melanoma cancer. Given its anti-tumor effect, there is a real hope that N-trimethyl chitosan-encapsulated camptothecin could serve as a novel and safe therapeutic option in the treatment of human melanoma.

## Competing interests

The authors declare that they have no competing interests.

## Authors' contributions

XPL carried out animal experiment, histological analysis, TUNEL staining, statistical analyses and drafted the manuscript. STZ carried out the MTT assay, flow cytometric analysis and revised the manuscript. XYL prepared the camptothecine nanoparticles and drafted the method of the preparation. XCC contributed to histological analysis and revised the manuscript. XZ participated in the design of the study, supervised experimental work and revised the manuscript. ZYQ offered camptothecine and nanoparticle, and participated in the preparation of the camptothecine nanoparticles. LNZ participated in animal experiment, histological analysis and TUNEL staining. ZYL contributed to animal experiment and TUNEL staining. YMW participated in statistical analyses. QZ, TY, ZYL and XH contributed to animal experiment. YQW conceived of the study and designed the topic. All authors read and approved the final manuscript.
